# Afatinib for the treatment of advanced non-small-cell lung cancer harboring an epidermal growth factor receptor exon 18 E709_T710delinsD mutation: a case report

**DOI:** 10.1186/s13256-021-02994-0

**Published:** 2021-11-22

**Authors:** Lander Van Acker, Dieter Stevens, Karim Vermaelen, Veerle Surmont

**Affiliations:** University Hospital Ghent, Ghent, Belgium

**Keywords:** NSCLC_1_, EGFR exon 18_2_, E709_T710delinsD mutation_3_, Afatinib_4_, Case report_5_

## Abstract

**Background:**

The discovery of epidermal growth factor receptor oncogenic driver mutations has changed the therapeutic landscape of advanced non-small cell lung cancer in the past decade. Since the introduction of next-generation sequencing, uncommon epidermal growth factor receptor mutations are more frequently discovered. Because seldom evaluated in clinical trials, their clinical significance and response on tyrosine kinase inhibitors are less well known.

**Case presentation:**

A 58-year-old Caucasian woman with no smoking history presented with advanced non-small cell lung cancer. Liver biopsy revealed an adenocarcinoma with a programmed death ligand-1 tumor proportion score of 30% and no common oncogenic driver mutations. A combination of chemotherapy and immunotherapy was started as first-line treatment. However, treatment was ceased after 18 weeks because of immune-related renal failure and disease progression. In the meantime, the next-generation sequencing results of the liver biopsy had revealed an exon 18 E709_T710delinsD mutation. Therefore, afatinib was administered, which was moderately tolerated with grade 2 paronychia and acneiform skin eruption. After 6 months, a partial response with ongoing decrease of the liver metastasis was retained.

**Conclusion:**

Because of the lack of clinical trials, tumor heterogeneity, and a tyrosine kinase inhibitor affinity related to the different mutation types, it is difficult to predict the clinical outcome of tyrosine kinase inhibitor in uncommon mutations. Therefore, a therapeutic trial with tyrosine kinase inhibitor has to be considered, but the expected clinical response is lower than for common mutations.

## Introduction

The discovery of epidermal growth factor receptor (EGFR) oncogenic driver mutations preluded a new therapeutic area for lung cancer treatment in the past decade. EGFR mutations are present in 30–60% of Asian and 10–20% of Caucasian patients with non-small-cell lung cancer (NSCLC). These mutations are more frequently observed in female patients, patients with lung adenocarcinoma, and never smokers. Three generations of tyrosine kinase inhibitors (TKI) have already been developed for common EGFR mutations and showed clinical efficacy in several phase III trials as first- and second-line treatment for metastatic non-small-cell lung adenocarcinoma [1, 2]. Recent data also revealed favorable disease-free survival with TKIs as an adjuvant treatment in resectable disease [3].

Afatinib, a second-generation TKI, has showed to improve the progression-free survival (PFS) in patients with common mutations such as exon 19 deletions and exon 21 Lco858R point mutations, accounting for approximately 85% of the EGFR mutations [4–6]. Its importance has decreased since the introduction of osimertinib with better results on PFS and toxicity in first-and second-line treatment [2, 7].

The clinical significance of uncommon EGFR mutations is less known. Few clinical data, mostly based on case reports, reported a favorable response on afatinib in EGFR exon 18 mutations [8]. We present a case report of a patient with advanced stage lung adenocarcinoma with an E709_T710delinsD mutation.

## Case presentation

**Fig. 1:**
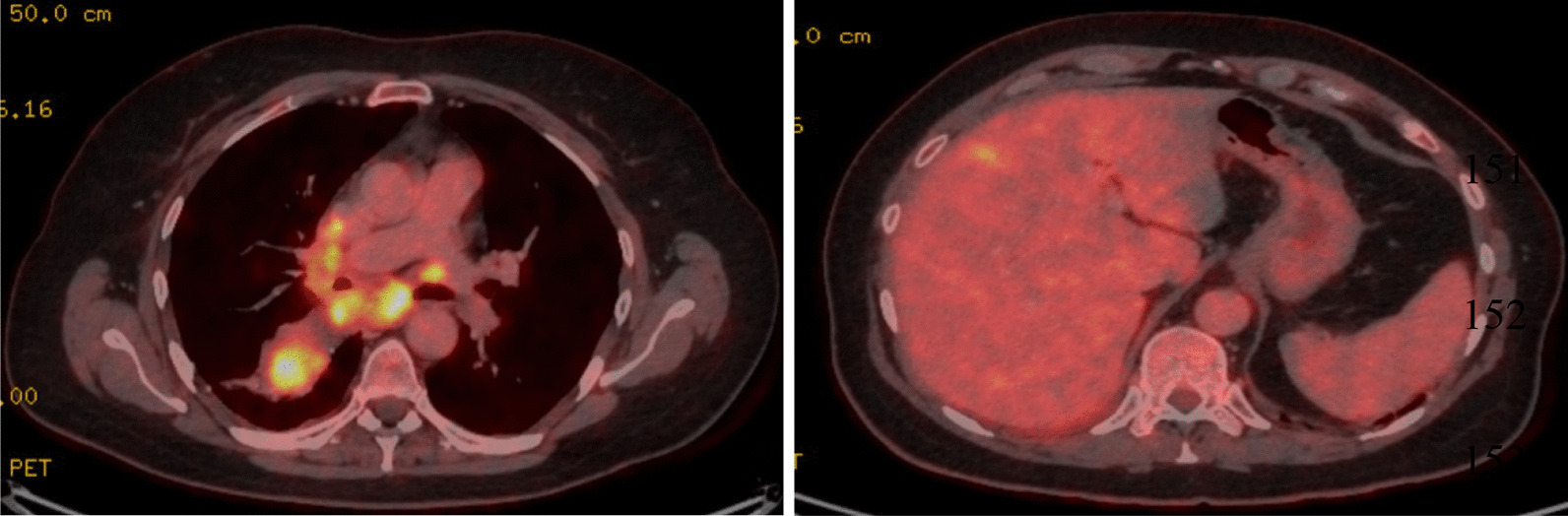
Positron Emission Tomography-Computed Tomography (PET-CT) at presentation

**Fig. 2:**
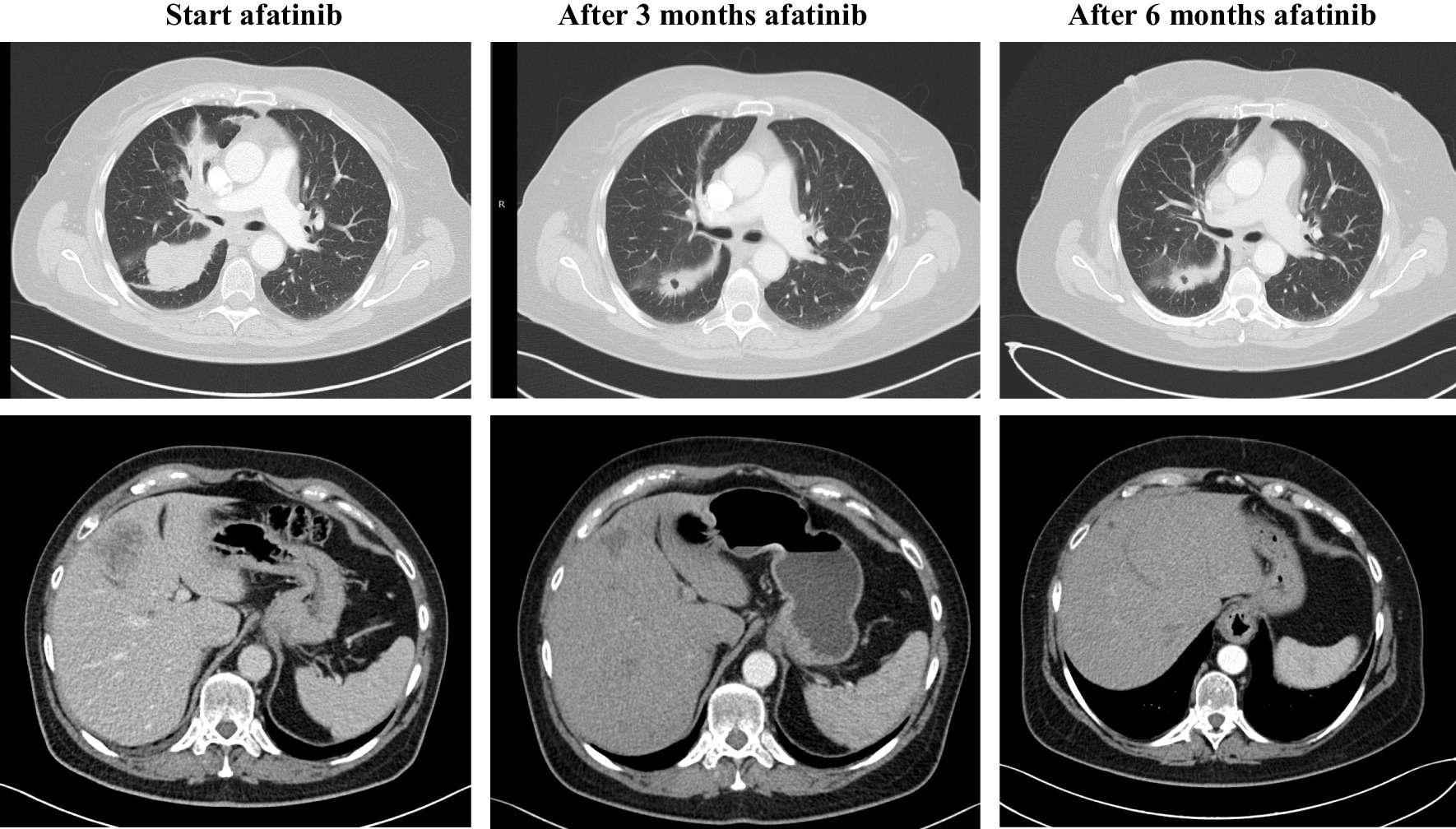
Radiographic response after afatinib. Standardized uptake value (SUV) lung lesion 10.8 g/ml

A 58-year-old Caucasian woman presented with a chronic cough and progressive dyspnea since 3 months. Her medical history included urolithiasis and a multinodular goiter, treated with a left hemithyroidectomy and thyroid hormone substitution. She had no antecedents of pulmonary disease, and there was no active or passive tobacco exposition. Her sister was treated for breast cancer, but no other family history of cancer was mentioned. Her World Health Organization (WHO) performance score was 1. Clinical examination revealed decreased breath sounds at the base of the right lung. Computed tomography (CT) revealed a necrotic mass in the apical segment of the right lower lobe, extending to the right hilum and encasing the right main and lower lobe bronchus. There were several enlarged mediastinal lymph nodes and right-sided pleural fluid. Positron emission tomography (PET) visualized a fluorodeoxyglucose (FDG)-avid liver lesion in segment 4b (Fig. 1). Pathologic diagnosis of an adenocarcinoma [thyroid transcription factor 1 (TTF1) positive] was confirmed by endobronchial ultrasound and CT-guided liver biopsy. The malign process was staged as a cT2aN3M1b or stage IVb adenocarcinoma (TNM 8th edition). The programmed death ligand-1 (PD-L1) tumor proportion score was 30% using the Roche SP 263 antibody assay. Anaplastic lymphoma kinase (ALK)- and reactive oxygen species 1 (ROS1)- immunohistochemistry was negative. Molecular analysis, performed by next-generation sequencing (NGS, SeqCap) revealed an EGFR mutation in exon 18, E709_T710delinsD. Since the result of the NGS was not yet available, the patient was included in a phase III randomized controlled trial with carboplatin, pemetrexed, and experimental immunotherapy with a combined transforming growth factor beta (TGF-β) and PD-L1 inhibitor. After two cycles, a partial response (PR) was observed with a stable situation after four cycles. Maintenance study immunotherapy was continued, but after two cycles there was disease progression of the primary tumor and the liver metastasis. Furthermore, the patient was hospitalized with grade 3 acute kidney injury, caused by an immune-mediated interstitial nephritis. The treatment was ceased, and renal function was restored after corticosteroid administration during 3 months. Two months later, afatinib 40 mg was initiated because of progressive disease (PD). This treatment was moderately tolerated with grade 2 paronychia and a grade 2 acneiform skin eruption, treated with tetracycline and topical therapy. After 6 months, a PR with ongoing decrease of the liver metastasis was retained (Fig. 2). Because of persistent grade 2 skin toxicity, the dose of afatinib was reduced to 30 mg.

## Discussion

The most common EGFR mutations are found in exons 19–21, with exon 19 deletions (45%) and exon 21 L858R point mutations (40%) being responsible for 85–90% of the EGFR-mutated NSCLC [6, 9, 10]. Inhibition of this mutated EGFR with the third-generation TKI osimertinib as first-line treatment resulted in an objective response rate (ORR) of about 80% of the patients, with a PFS of 18.9 months and an overall survival (OS) of 38.6 months in the FLAURA trial. T790M (exon 20) is a rather uncommon mutation, but its clinical significance is well known as the cause of TKI acquired resistance in almost 50–60% of the patients after first- or second-line TKI treatment [2, 11].

The other, so-called uncommon, mutations are present in 7–23% of the EGFR-mutated NSCLC. They are more heterogeneous (about 600 known variations), and the detection of new variants is still ongoing. Exon 20 insertions (6%), exon 18 G719X (3%), exon 21 L861Q (1%), S768I (1%), and exon 19 insertions (0.6%) are more frequently reported mutations, noted as major uncommon mutations [8]. They are less gender dependent and also occur in (ex-)smokers [11, 12]. However, the prevalence of uncommon mutations will probably change in the next coming years, due to the broader application of NGS for molecular tumor profiling. Until recently, EGFR mutation testing was mostly based on commercial genotyping assays (COBAS, Therascreen) with heterogenic targets. For example, most kits only included exon 18 G719 point mutations and no E709_T70delinsD mutations [8].

Exon 18 mutations account for 4.1% of all EGFR mutations following the catalogue of somatic mutations in cancer (COSMIC) database [11, 13]. In a large French study, a frequency of 5.5% was found [14]. Two types are described: a deletion–insertion or a point mutation. G719X (mostly G719A), followed by E709K, were the more frequently observed point mutations. E709-T710delinsD was the most common deletion, but only accounting for less than 0.1% of the EGFR mutations [9, 15]. Seventeen cases of an E709_T70delinsD mutation have been reported in the COSMIC database and 27 in MyCancergenome.org [13, 16].

Compound mutations, consisting of a common and an uncommon mutation, are more frequent than single mutations, harboring 2–25% of the EGFR mutation-positive lung cancers. The prognosis of patients with these mutations is reduced in comparison with two common mutations, but remains higher than for uncommon mutations [6].

Less is known about the clinical relevance and therapeutic response when targeting single uncommon mutations because they were excluded in most trials. Moreover, there are no specific clinical trials exploring the effect of TKIs on EGFR exon 18 mutations. Small numbers of patients with uncommon mutations (+/− 10%) were included in trials with gefitinib (Iressa Pan-Asia Study) and afatinib (LUX-Lung 3 and 6), but the predictive value could not be determined owing to the small study populations [8, 11]. In a post hoc analysis of afatinib, performed by Yang *et al.*, there was demonstrated clinical activity against the major uncommon mutations (exon 18 G719X, exon 21 L861Q, and S768I). The response rate for the sensitizing exon 18 G719X mutations was lower than for common mutations, but exceeded 50% (63.4%) with a time to treatment failure of 14.7 months (8.1–17.1 months) in treatment-naïve patients [8]. Recently, some evidence for osimertinib in uncommon mutations was published by Cho *et al.* For example, an objective response of 53% with a PFS of 8.2 months was found for exon 18 G179X mutations [17].

In a French retrospective study, the PFS for exon 18 mutations after TKI was 14.6 months against 5.8 months after chemotherapy. The OS was 12.2 months, influenced by the fact that one-third of the people did not manage to receive a TKI after chemotherapy. In conclusion, the authors proposed a trial with TKI as first-line therapy. However, the therapeutic response was very heterogeneous among the mutation variants and higher for proximal localized mutations (G719X and E709X point mutations), so the effect remained difficult to predict [14].

There are even fewer data available for the specific exon 18 E709_T70delinsD mutation [8]. An *in vitro* study, performed by Kobayashi *et al.* explored the role of exon 18 mutations, inclusive E709_T70delinsD, and appointed these as oncogenic driver mutations. They observed a higher sensitivity for second-generation TKIs in comparison with first- and third-generation TKIs [9]. The underlying mechanism seemed to be a variable affinity to the targeted kinase, with the highest affinity for afatinib [8, 9].

Because of scarce evidence, treatment responses of afatinib in patients with an E709_T70delinsD mutation are mostly based on several case series. On the website https://www.uncommonegfrmutations.com clinical cases are collected to get a better insight in the clinical outcome. An overview of the published case reports in literature is presented in Table 1 with a total of 14 cases. All patients had a history of an adenocarcinoma without a gender predominance. They were more frequent of Asian origin, and most of them had no tobacco history. Seven patients (50%), including all cases with afatinib, had a PR. One case showed a good intracranial response, although combined with bevacizumab. Data of OS are lacking and are difficult to compare because patients had undergone multiple previous treatment lines [4, 9, 10, 14, 15, 18–22].

**Table 1:** Summary of the case reports with an epidermal growth factor receptor exon 18 E709_T710delinsD [4, 9, 10, 14, 15, 18–22]

Case report	Ethnicity	Age	Gender	Smoking	Histology	TKI	Response	PFS (m)	OS (m)
Wu *et al*.	Asian	61	F	No	AD	Gefitinib	SD	5.1	22.7
Wu *et al*.	Asian	65	M	Yes	AD	Gefitinib	PD	0.9	11.1
Kobayashi *et al*.	Asian	63	M	N/A	AD	Erlotinib** Afatinib	SD PR	N/A N/A	N/AN/A
Ackerman *et al*.	Non-Asian	88	F	No	AD	Erlotinib	PR	6 (stop)	N/A
Leduc *et al*.	Non-Asian	N/A	N/A	N/A	N/A	N/A	N/A	3	N/A
Ibrahim *et al*.	Non-Asian	52	F	No	AD	Afatinib	PR	N/A	N/A
Iwamoto *et al*.	Asian	56	F	No	AD	Afatinib	PR	7	N/A
An *et al*.	Asian	71	M	Yes	AD	Afatinib***	PR	11	> 21
Klughammer *et al*.	Asian	50	F	No	AD	Erlotinib*	PD	38	52
Mehta *et al*.	Asian	61	M	N/A	AD	Erlotinib*	PR	7	N/A
Sousa *et al*.	Non-Asian	N/A	N/A	N/A	N/A	Gefitinib	PD	3	24
Sousa *et al*.	Non-Asian	N/A	N/A	N/A	N/A	Erlotinib*	PD	4	26
Sousa *et al*.	Non-Asian	N/A	N/A	N/A	N/A	Erlotinib*	PD	3	18
This case	Non-Asian	58	F	No	AD	Afatinib*	PR	> 6	N/A

*AD* adenocarcinoma, *PR* partial response, *SD* stable disease, *PD* progressive disease, *N/A* not available, *second-line treatment; **intolerance; ***+ bevacizumab

## Conclusion

This case report is an example of a favorable response in an EGFR-mutated NSCLC with a “minor” uncommon mutation successfully treated with afatinib. Because of the limited evidence and the absence of clinical trials, no robust treatment recommendations can be made. EGFR-TKI therapy, with most reports available for afatinib, can be an option in patients with exon 18 mutations, but the expected clinical response is lower than for common mutations. However, tumor heterogeneity and a TKI affinity related to the different mutation types complicate the prediction of the clinical outcome of TKI in uncommon mutations. In conclusion, a therapeutic trial with TKI has to be considered.

## Data Availability

Not applicable.

## Abbreviations

EGFR: Epidermal growth factor receptor
NSCLC: Non-small-cell lung cancer
NGS: Next-generation sequencing
TKI: Tyrosine kinase inhibitor
PD-L1: Programmed death ligand 1
PFS: Progression-free survival
WHO: World Health Organization
CT: Computed tomography
PET: Positron emission tomography
FDG: Fluorodeoxyglucose
TTF1: Thyroid transcription factor 1
ALK: Anaplastic lymphoma kinase
ROS1: Reactive oxygen species 1
PR: Partial response
PD: Progressive disease
ORR: Objective response rate
OS: Overall survival
COSMIC: Catalogue of somatic mutations in cancer
